# Supplementary material for: Single-cell multiomics identifies both shared and unique features of immune dysfunction in Parkinson’s disease and inflammatory bowel disease colon, plasma and stool
Source: bioRxiv. 2025 Jun 11:2025.04.29.651228. Preprint. [Version 2] doi: 10.1101/2025.04.29.651228 (PMC12258910; doi:10.1101/2025.04.29.651228)
Supplement: Supplement 1 [file NIHPP2025.04.29.651228v2-supplement-1.pdf]

bioRxiv preprint doi: https://doi.org/10.1101/2025.04.29.651228; this version posted June 11, 2025. The copyright holder for this preprint (which was not certified by peer review) is the author/funder, who has granted bioRxiv a license to display the preprint in perpetuity. It is made available under aCC-BY 4.0 International license.

Supplemental Table 1. Colonic biopsy patient demographics and cognitive status

	PD<br>N= 12	IBD<br>N= 13	NHC<br>N= 9
**Sex F/M**	2/10	6/7	4/5
**Race/Ethnicity**	11 White	13 White	9 White
**NSAID use /never use**	4/8	8/5	7/2
**Age**<br>Avg ± SD<br>Median (IQR)	62.7 ± 8.8<br>63 (11)	57.08 ± 7.8<br>58 (8.5)	62.4 ± 8.3<br>65 (12.5)
**Disease Duration**<br>Avg ± SD<br>Median (IQR)	5.55 ± 4.2<br>4 (7)	N/A	N/A
**MoCA Score**<br>Avg ± SD<br>Median (IQR)	25.64 ± 3.9<br>26 (8)	N/A	N/A
**Schwab and England ADL Scale**<br>Avg ± SD<br>Median (IQR)	90.09 ± 7.7<br>90 (5)	N/A	N/A

MoCA (Montreal Cognitive Assessment) Score: score reflecting the degree of cognitive impairment and signs of early dementia (score>26=normal, score<26=cognitive impairment).
Schwab and England Activities of Daily Living Scale: scale reflecting an individual's ability to function in activities of daily living (100%=very independent, 50%=very dependent, 0%=bed-ridden).
IQR: Interquartile range

bioRxiv preprint doi: https://doi.org/10.1101/2025.04.29.651228; this version posted June 11, 2025. The copyright holder for this preprint (which was not certified by peer review) is the author/funder, who has granted bioRxiv a license to display the preprint in perpetuity. It is made available under aCC-BY 4.0 International license.

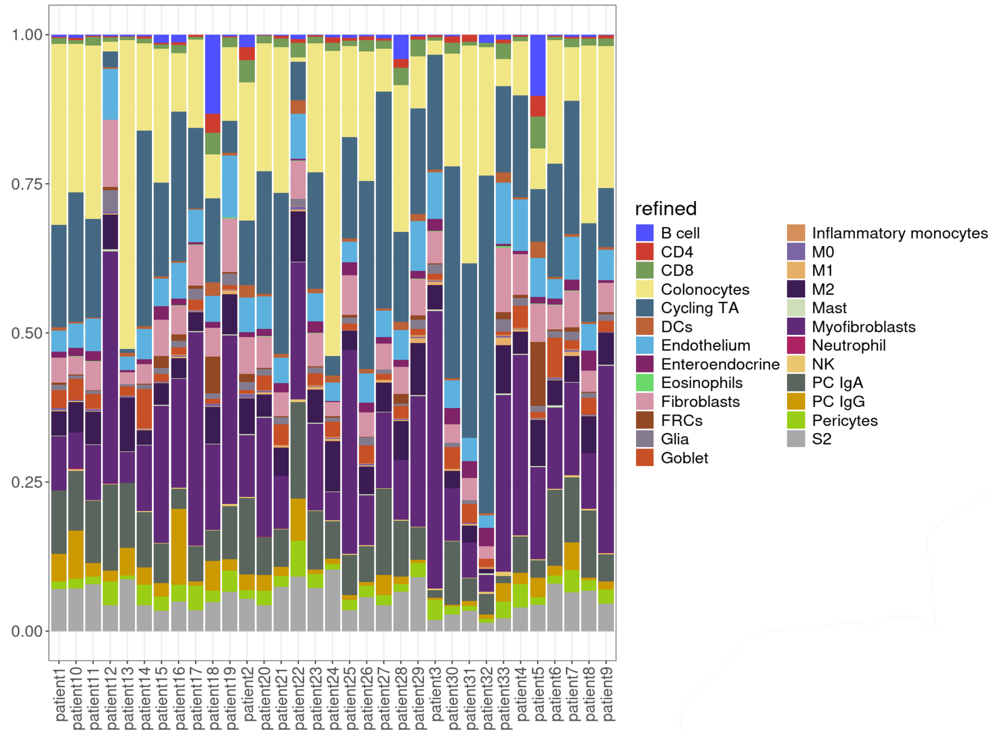

Supplemental Figure 1. Refined cell-type abundance from every participant.

bioRxiv preprint doi: https://doi.org/10.1101/2025.04.29.651228; this version posted June 11, 2025. The copyright holder for this preprint (which was not certified by peer review) is the author/funder, who has granted bioRxiv a license to display the preprint in perpetuity. It is made available under aCC-BY 4.0 International license.

Supplemental Table 2. CosMx SMI gene cluster identifiers.

Cell-type	Subpopulation Markers	Refined population markers
Epithelial cells	EPCAM, AQP8, BEST4, MUC2, OLFM4, PLCG2, TRPM5, ZG16	Colonocytes: AQP8, FABP1 and SLC26A2<br>Goblet cells: MUC2, TFF3 and SPINK4<br>Enteroendocrine: CHGA<br>Cycling TA: MKI67, TOP2A and PCNA
B cells	CD79A, BANK1, CD19, DERL3, MS4A1, MZB1	NA
Plasma cells	DERL3, MZB1, XBP1	PC IgA: +IgA and DERL3, MZB1, XBP1, -IgG<br>PC IgG: +IgG and DERL3, MZB1, XBP1, -IgA
T cells	CD3D, CD3E, CD3G, CD8A, FOXP3, GZMA, GZMB, IL17A, NKG7, TRBC1	CD8: CD8A, CD8B, GZMK, KLRB1, SPRY1 and IGTA1 and KLRG1<br>CD4: CD4, CCR7, LEF1, ANXA1, IL7R and GPR186<br>and SELL<br>NK: KLRF1, NCAM1, and lacks expression of CD3G, CD3D, CD8A and CD4
Stromal cells	ACTA2, ADAMDEC1, CHI3L1, COL3A1, NRXN1, PVALP, SOX6, VWF	S2: VSTM2, NPY and NRG1<br>Endothelium: VWF, PECAM1 and PLVAP<br>Fibroblasts: ADAMDEC1, CP, OGN, CCDC80 and GREM1<br>and FABP4<br>FRCs: CCL19 and CCL21<br>Glia: NRXN1, S100B and CDH19<br>Myofibroblast: SOSTDC1, ACTG2 and MYH11<br>Pericyte: NOTCH3 and RGS5
Myeloid cells	AIF1, C1QA, C1QB, CD14, CMTM2, FCGR3B, LYZ, MS4A2, TPSAB1, TPSAB2	M0: lack the expression of M2 markers and expression of CD68, C1QA, C1QB, SELENOP, LYZ, HLA-DPB1, HLA-DPA1, AIF1<br>M1: ACOD1,TNIP3, IL1B, INHBA, IL6, VCAN, CD300E, CXCL5, TNIP3, IL1B, INHBA,<br>IL6, VCAN, CD300E<br>M2: CD209, CD163L1 and FOLR2<br>Inflammatory monocytes: VCAN, CD300E, CD14, FCN1<br>and S100A9<br>Mast cells: LTC4S, TPSB2 and TPSAB1<br>Eosinophils: CLC, IL4 and IL13<br>Neutrophils: PROK2, CMTM2, CXCL8, FCRG3B, AQP9, S100A8 and S100A9<br>DCs: CD1C, TRL10 , TCTN3<br>CCL22 , CCL19, LAMP3

bioRxiv preprint doi: https://doi.org/10.1101/2025.04.29.651228; this version posted June 11, 2025. The copyright holder for this preprint (which was not certified by peer review) is the author/funder, who has granted bioRxiv a license to display the preprint in perpetuity. It is made available under aCC-BY 4.0 International license.

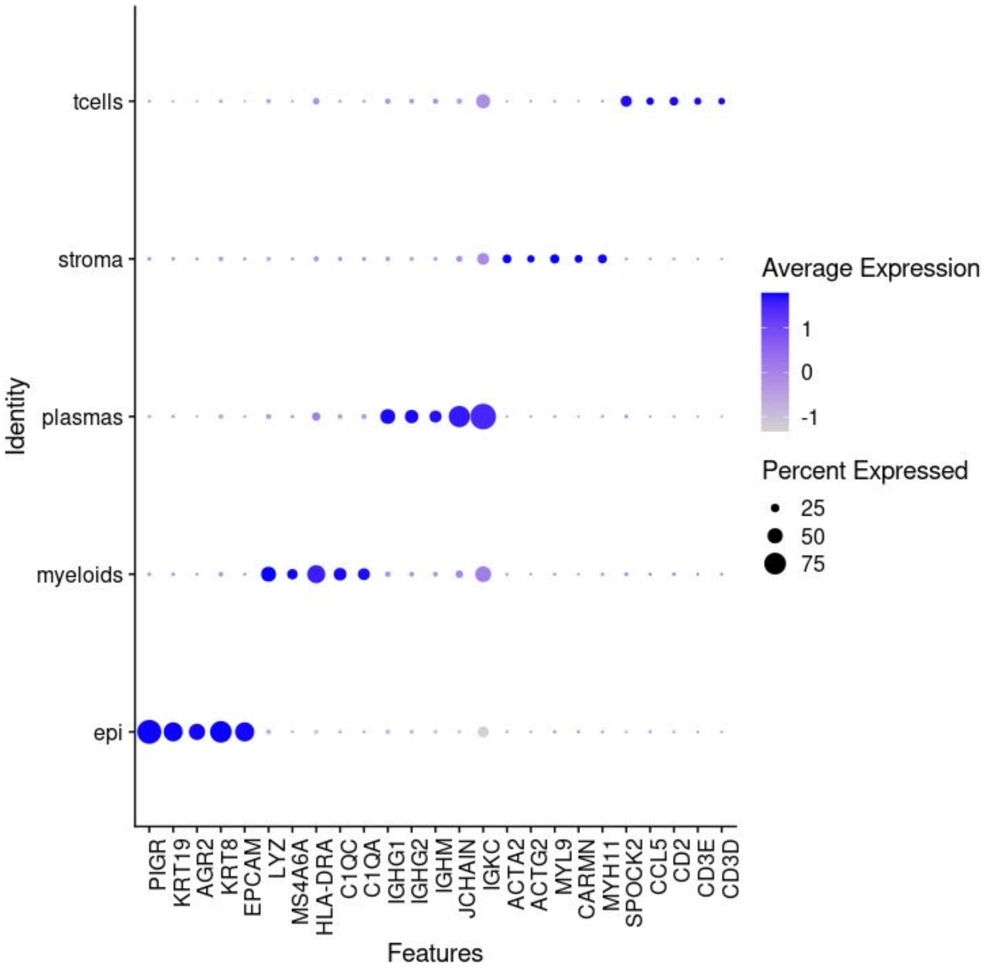

Supplemental Figure 3. CosMX SMI transcriptomics Cell cluster identifiers.

bioRxiv preprint doi: https://doi.org/10.1101/2025.04.29.651228; this version posted June 11, 2025. The copyright holder for this preprint (which was not certified by peer review) is the author/funder, who has granted bioRxiv a license to display the preprint in perpetuity. It is made available under aCC-BY 4.0 International license.

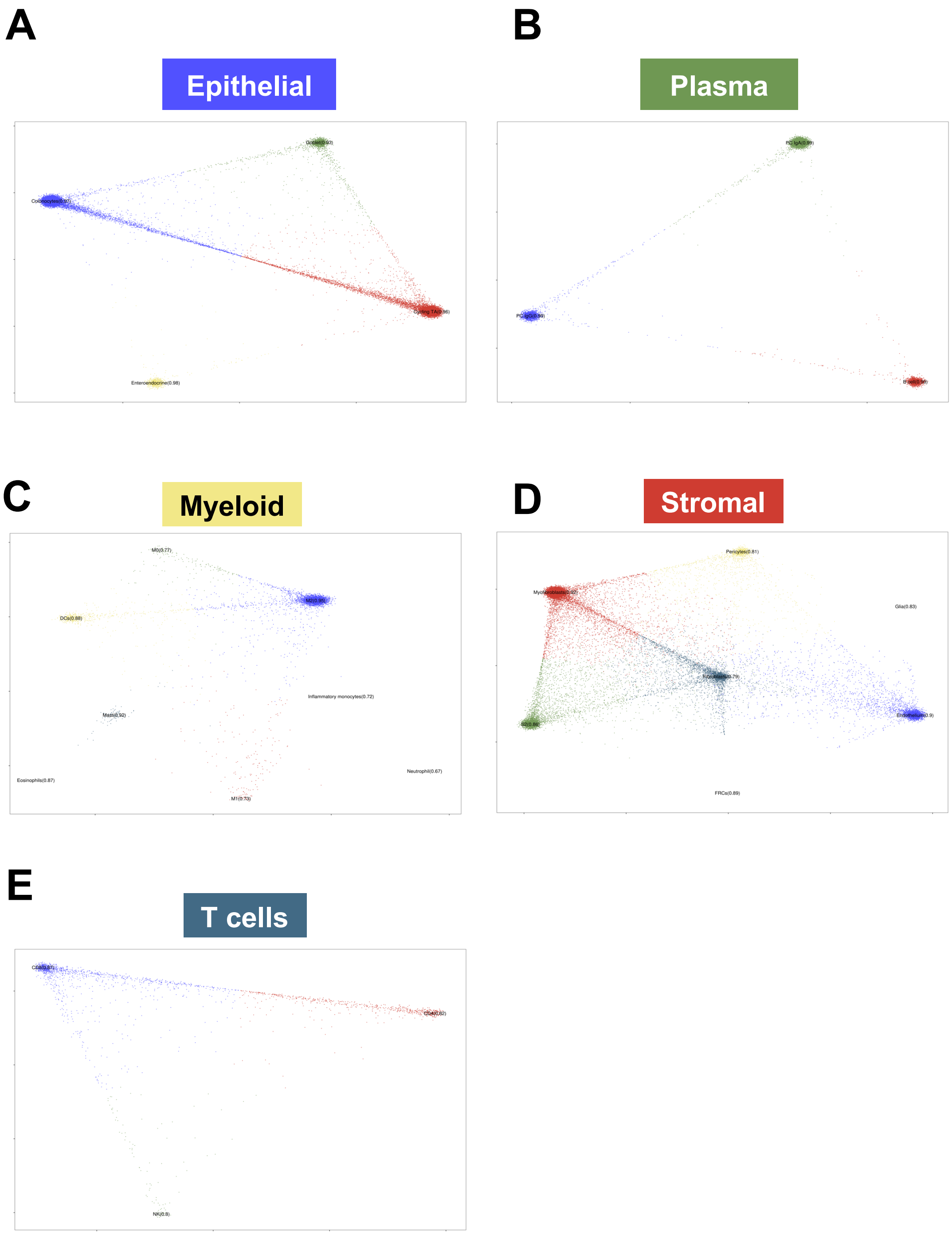

Supplemental Figure 4. CosMx SMI refined gene cluster flight-path analysis.

bioRxiv preprint doi: https://doi.org/10.1101/2025.04.29.651228; this version posted June 11, 2025. The copyright holder for this preprint (which was not certified by peer review) is the author/funder, who has granted bioRxiv a license to display the preprint in perpetuity. It is made available under aCC-BY 4.0 International license.

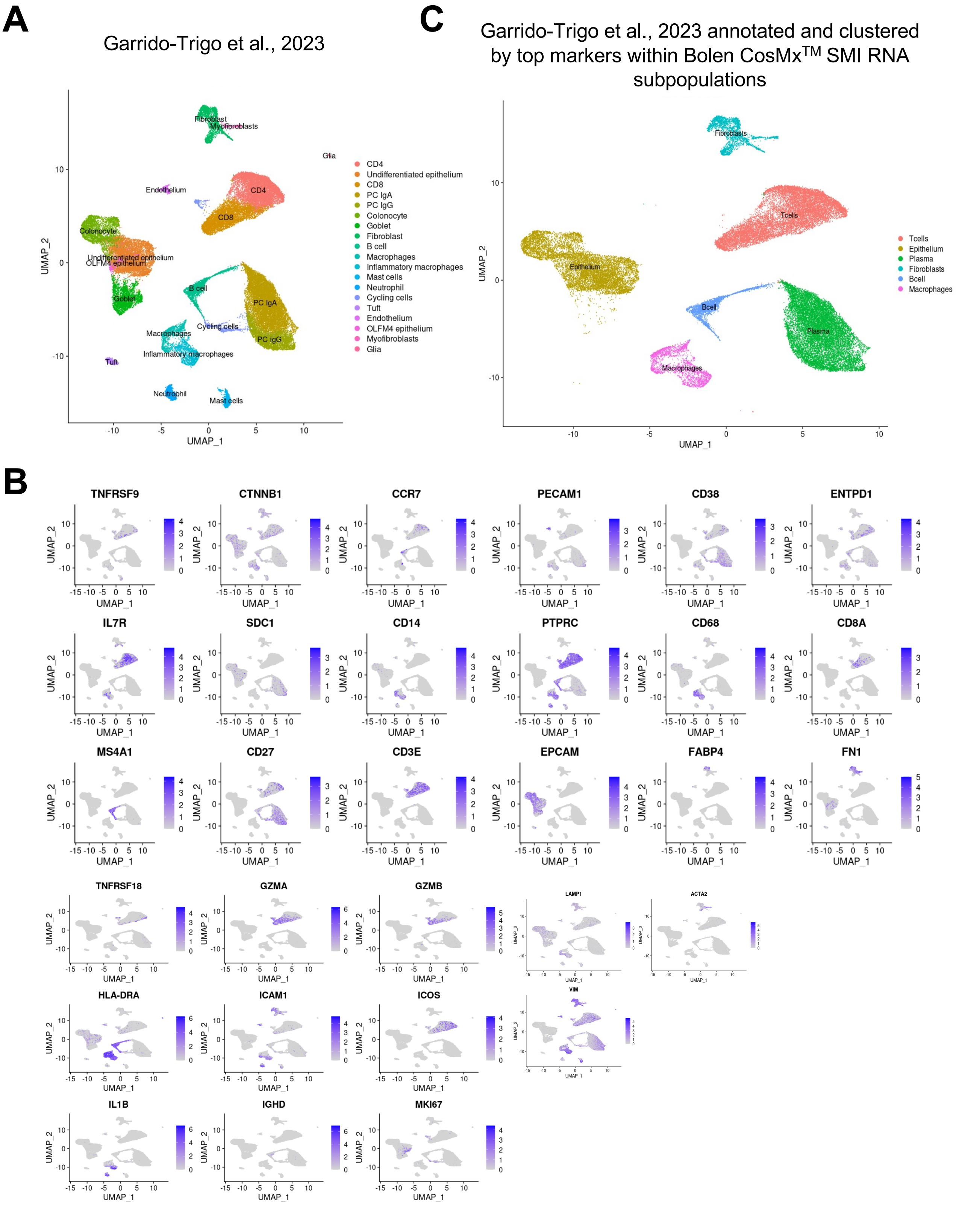

Supplemental Figure 5. Single-cell targeted proteomic analysis successfully integrates with scRNA-seq and depicts multimodal immune dysregulation across multiple biological outputs of human colonic biopsies

bioRxiv preprint doi: https://doi.org/10.1101/2025.04.29.651228; this version posted June 11, 2025. The copyright holder for this preprint (which was not certified by peer review) is the author/funder, who has granted bioRxiv a license to display the preprint in perpetuity. It is made available under aCC-BY 4.0 International license.

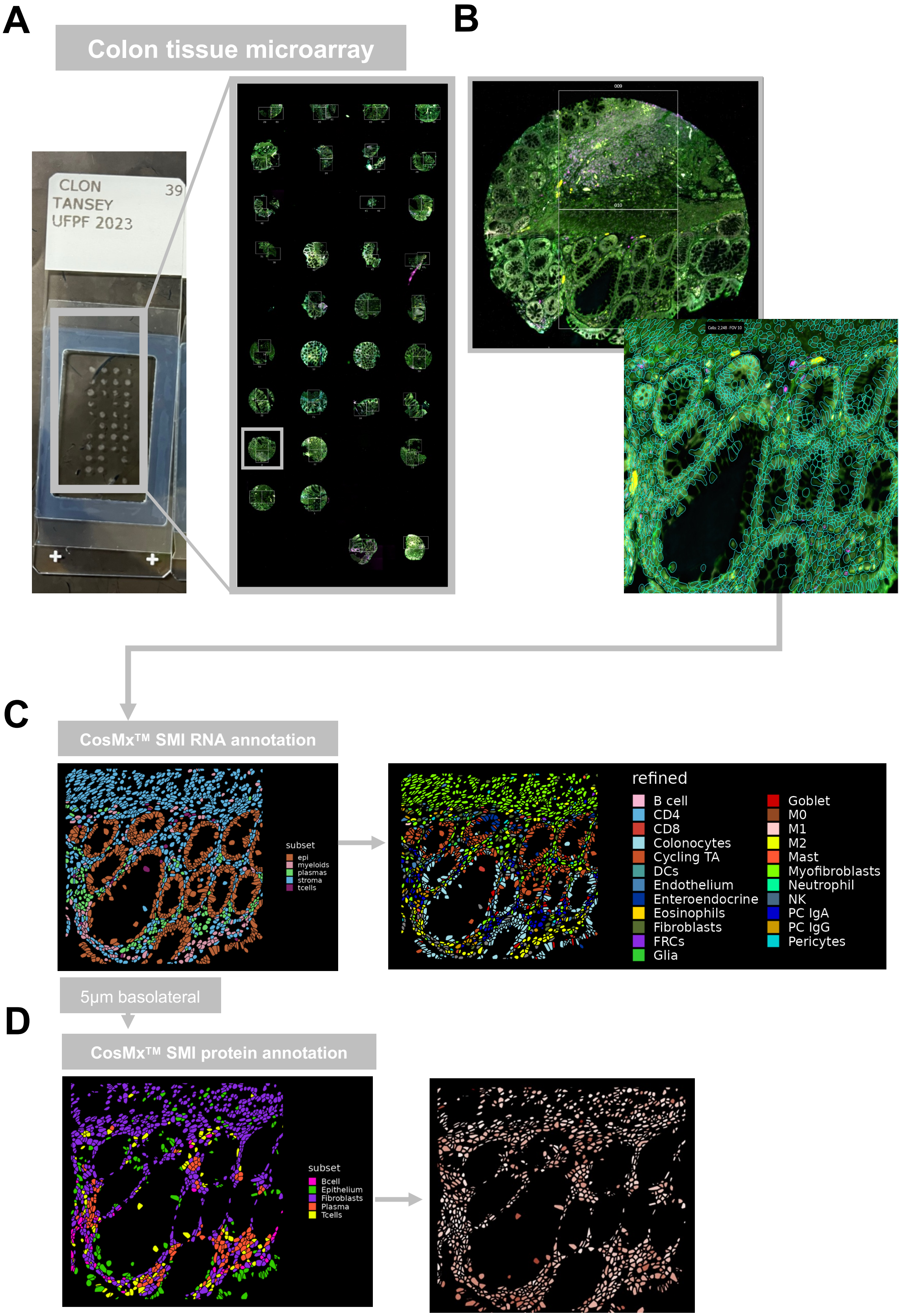

Supplemental Figure 6. Analysis workflow of CosMx^{TM} SMI RNA and protein analyses.

bioRxiv preprint doi: https://doi.org/10.1101/2025.04.29.651228; this version posted June 11, 2025. The copyright holder for this preprint (which was not certified by peer review) is the author/funder, who has granted bioRxiv a license to display the preprint in perpetuity. It is made available under aCC-BY 4.0 International license.

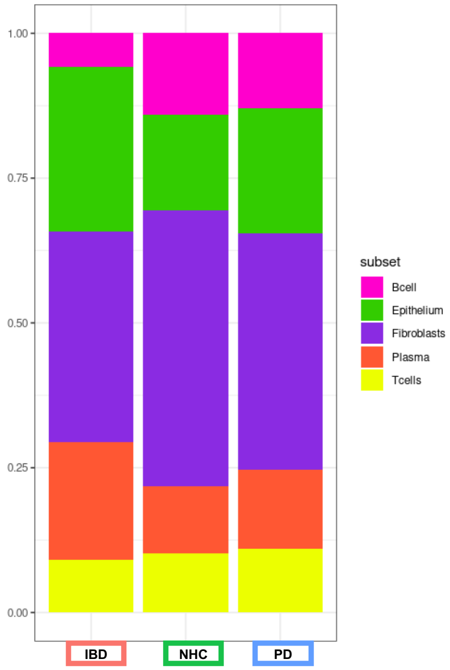

Supplemental Figure 7. Spatial molecular imaging (SMI) of directed proteome provides cell-type specificity and frequency at the level of subpopulations in sigmoid colon biopsies from those living with inflammatory bowel disease in remission (IBD) and Parkinson's disease (PD).
